# Short-term outcomes of laparoscopic versus open major hepatectomy for benign liver disease: an international bicentric study

**DOI:** 10.1007/s00423-026-04028-z

**Published:** 2026-04-10

**Authors:** Maciana Santos Silva, Gilton Marques Fonseca, Adriano C. Costa, Alessandro Mazzotta, Vagner Birk Jeismann, Jaime Arthur Pirola Kruger, Wellington Andraus, Fabricio Ferreira Coelho, Brice Gayet, Olivier Soubrane, Paulo Herman

**Affiliations:** 1 Department of Gastroenterology, Hospital das Clínicas da Faculdade de Medicina da Universidade de São Paulo, Av. Dr. Enéas de Carvalho de Aguiar, 255, 9th Floor, Room 9074. Cerqueira César, São Paulo, São Paulo, 05403- 000 SP France; 2 Department of Digestive, Metabolic and Oncologic Surgery, Institut Mutualiste Montsouris, University René Descartes Paris 5, Paris, France

**Keywords:** Major hepatectomy, Laparoscopy, Benign liver disease, Minimally invasive surgery, Perioperative outcomes

## Abstract

**Purpose:**

Laparoscopic hepatectomy is well established for minor resections in benign liver disease; however, evidence for its reproducibility in major hepatectomy remains limited. Therefore, the purpose of this international bicentric study was to compare perioperative outcomes of laparoscopic versus open major hepatectomy for benign liver disease in two high-volume hepatobiliary centers.

**Methods:**

This retrospective international bicentric study analyzed prospectively maintained databases from two high-volume hepatobiliary centers in São Paulo, Brazil, and Paris, France, between 2000 and 2024. Adult patients undergoing right or left major hepatectomy for benign liver disease were included. Perioperative outcomes were compared between laparoscopic and open approaches using standardized definitions. Multivariate analysis identified independent predictors of intraoperative transfusion and early hospital discharge.

**Results:**

A total of 126 patients were included (78 open; 48 laparoscopic). Laparoscopic major hepatectomy was associated with lower blood loss (150 vs. 400 mL, *p* < 0.001), reduced transfusion rates (8.3% vs. 38.5%, *p* = 0.001), shorter hospital stay (5 vs. 7 days, *p* < 0.001), and fewer biliary leakage (4.2% vs. 20.5%, *p* = 0.016). On multivariate analysis, the laparoscopic approach independently predicted lower transfusion risk (OR 0.096, 95% CI 0.011–0.862; *p* = 0.036) and early hospital discharge (OR 0.083, 95% CI 0.008–0.847; *p* = 0.036). Operative time, ICU admission, overall morbidity, and mortality were comparable between approaches, with no postoperative deaths.

**Conclusion:**

Laparoscopic major hepatectomy for benign liver disease is safe and feasible in experienced centers and is associated with improved short-term perioperative outcomes compared with the open approach.

## Introduction

Laparoscopic liver resection (LLR) has advanced substantially since its introduction in the early 1990s, particularly for peripheral and benign lesions, and is now considered the gold standard for selected minor resections [1–3]. Minimally invasive liver surgery offers well-established advantages, including reduced postoperative pain, faster recovery, shorter hospital stay, and lower morbidity when compared with open hepatectomy [2–5]. However, the role of LLR in major hepatectomy, defined as resection of three or more contiguous segments, remains less clearly characterized due to technical challenges, steep learning curves, and the limited availability of comparative international data, particularly for benign disease.

Benign liver lesions comprise a heterogeneous group of conditions in which surgery is generally reserved for symptomatic patients, those at risk of complications, or when diagnosis remains uncertain [6–8]. Therefore, the indication for resection should be guided by clinical criteria rather than by the availability of laparoscopic techniques. In selected patients, however, minimally invasive major hepatectomy may offer meaningful advantages, including reduced blood loss, decreased surgical trauma, attenuated inflammatory response, and enhanced postoperative recovery [9–11].

Despite these potential benefits, international data indicate that minimally invasive major hepatectomy still represents a minority of major liver resections in Europe [12], and that a substantial proportion of hepatobiliary surgeons worldwide have limited or no experience with this approach [13]. Consequently, most published series originate from experienced, high-volume centers and predominantly focus on malignant indications, such as colorectal liver metastases or hepatocellular carcinoma, limiting the generalizability of available evidence for benign disease.

The aim of this international bicentric study was to compare short-term perioperative outcomes of laparoscopic versus open major hepatectomy for benign liver disease in two high-volume hepatobiliary centers.

## Materials and methods

The Institutional Ethics Committee approved this protocol. This study was conducted in accordance with the Strengthening the Reporting of Observational Studies in Epidemiology (STROBE) recommendations [14].

A total of 2,587 liver resections performed between February 2000 and December 2024 were retrospectively reviewed from two prospectively maintained institutional databases at high-volume hepatobiliary centers (University of São Paulo, Brazil; Institut Mutualiste Montsouris, Paris, France). Among these, 145 patients underwent major hepatectomy for benign liver disease.

**Fig. 1:**
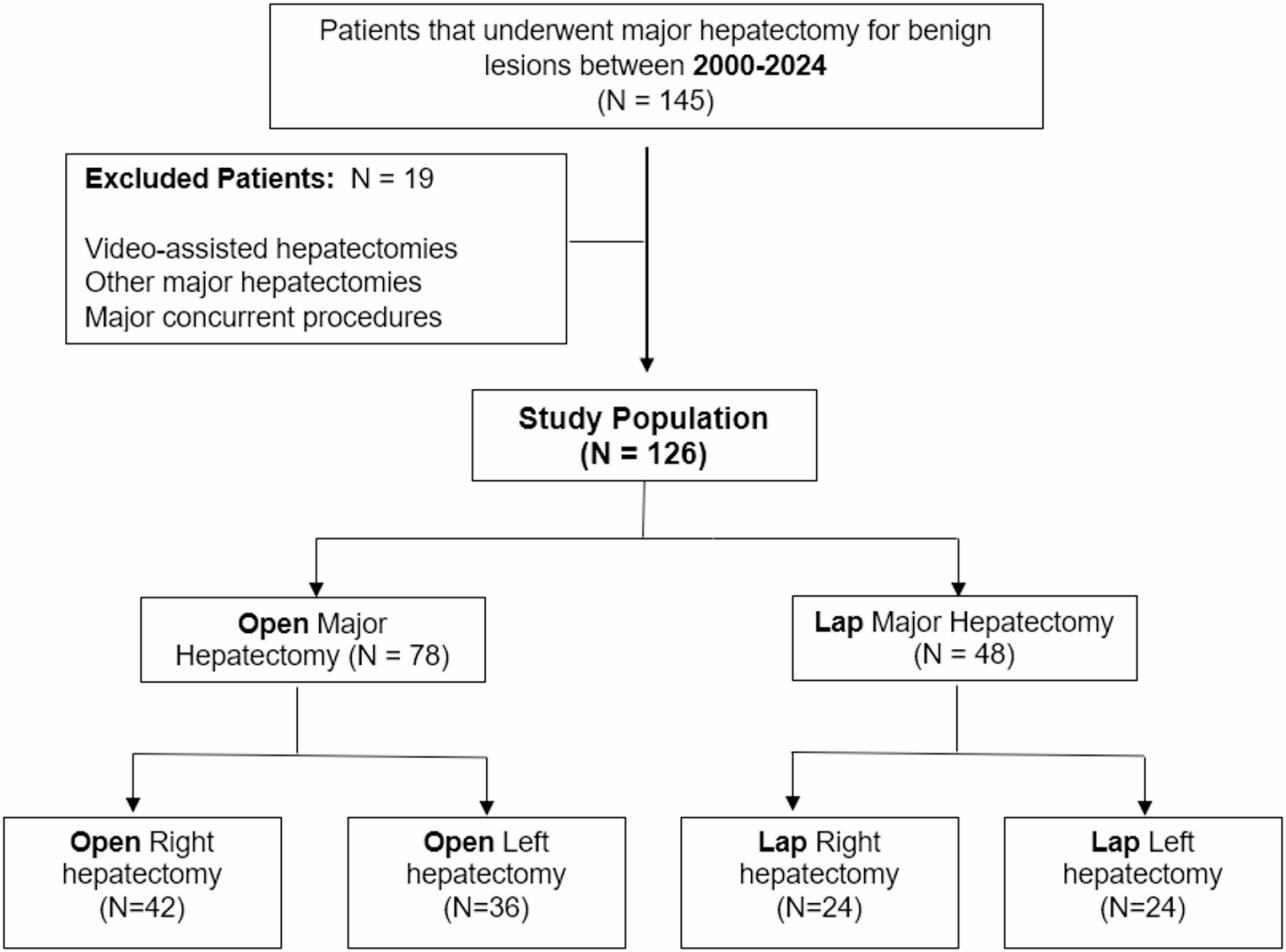
Study flowchart (attached image)

After applying predefined inclusion and exclusion criteria, 19 patients were excluded, resulting in a final cohort of 126 patients allocated to laparoscopic or open surgery groups (Fig. 1).

Patients were excluded if they had incomplete clinical data, previous liver surgery, malignant liver tumors, hybrid or hand-assisted laparoscopic procedures (*n* = 2), or underwent non-standardized resections not comparable between groups, such as trisectionectomies or atypical extended resections (*n* = 9). Patients undergoing simultaneous major procedures were also excluded, including biliodigestive anastomosis (*n* = 5), pancreatoduodenectomy (*n* = 1), gastric resection (*n* = 1), or gastrointestinal reconstruction (*n* = 1).

Only major right and left hepatectomies were included, as these standardized anatomical resections share comparable technical complexity and allow reliable comparison between surgical approaches. Patients were categorized according to surgical technique (laparoscopic vs. open), and further stratified by right or left major hepatectomy.

Major hepatectomy was defined according to the Brisbane classification [15]. Preoperative assessment included computed tomography and/or magnetic resonance imaging. All cases were discussed in multidisciplinary meetings, and all procedures were performed with curative intent. The choice of surgical approach reflected surgeon expertise and patient-specific anatomical and clinical considerations, evolving over time with increasing institutional experience.

### Variables and outcomes

Demographic, clinical, and preoperative variables were prospectively collected. Intraoperative variables and postoperative outcomes were compared between groups to assess perioperative performance.

Analyzed variables included operative time, intraoperative blood loss, use and duration of the Pringle maneuver, conversion to open surgery, intraoperative transfusion, postoperative complications (Clavien–Dindo classification), post-hepatectomy liver failure, ascites, bile leakage, ileus, acute kidney injury (AKI), intra-abdominal collections, ICU admission, total hospital stay, and 90-day mortality. Pathology reports were reviewed to confirm benign diagnosis and assess surgical margins.

Abdominal drains were not routinely placed after hepatectomy and were used selectively based on intraoperative findings, including lesion size, extent of the raw liver surface, and suspicion of bile leakage during surgical procedure. Postoperative computed tomography was not routinely performed before discharge and was reserved for patients in whom postoperative complications were clinically suspected.

Postoperative complications were graded according to the Clavien–Dindo classification [16]. Prolonged postoperative ileus was defined as gastrointestinal dysfunction requiring nasogastric decompression for more than three days [17]. AKI was defined according to Kidney Disease: Improving Global Outcomes (KDIGO) criteria [18]. Ascites was defined as abdominal drainage exceeding 10 mL/kg/day after postoperative day 3. Post-hepatectomy liver failure was defined according to the criteria of the International Study Group of Liver Surgery (ISGLS), namely elevated PT/INR associated with concomitant hyperbilirubinemia on or after postoperative day 5 [19]. Bile leakage was defined according to ISGLS criteria as drain bilirubin levels at least three times the serum concentration on postoperative day 3 [20].

Short-term outcomes were defined as perioperative events occurring during the index hospitalization and within the first 90 postoperative days. Readmissions within the first 30 postoperative days were also recorded.

### Surgical technique

Laparoscopic hepatectomy was performed using a five- or six-port technique with pneumoperitoneum maintained at 12 mmHg. Parenchymal transection was carried out using bipolar coagulation, ultrasonic shears, and/or CUSA, as appropriate. Major vascular structures were divided using clips or advanced energy-sealing devices. Specimens were extracted through a suprapubic incision, and abdominal drains were placed selectively based on intraoperative findings.

Open hepatectomy was performed through right subcostal (“J”), bilateral subcostal, or Mercedes-type incisions, following standard hepatobiliary surgical principles.

When required, hepatic inflow occlusion was performed using the Pringle maneuver, consisting of temporary clamping of the hepatoduodenal ligament with a vascular clamp or tourniquet. Intermittent clamping was typically applied using cycles of 15 min of occlusion followed by 5 min of reperfusion, depending on intraoperative bleeding and surgeon preference. In selected cases, a hemi-Pringle maneuver was performed, consisting of selective inflow occlusion of the ipsilateral portal pedicle supplying the hemiliver being resected, allowing targeted vascular control during parenchymal transection [21].

### Statistical analysis

Continuous variables were expressed as medians with interquartile ranges (IQR) and compared using the Mann–Whitney U test. Categorical variables were compared using the Chi-square or Fisher’s exact test, as appropriate. Multivariate logistic regression analysis was performed to identify independent predictors of intraoperative transfusion and early hospital discharge (≤ 6 days). A p-value < 0.05 was considered statistically significant. Statistical analyses were conducted using SPSS version 27 and R version 4.4.0.

Propensity score matching was not applied, as baseline demographic and clinical characteristics were well balanced between groups, allowing direct comparison of perioperative outcomes.

## Results

A total of 126 patients underwent major hepatectomy for benign liver disease during the study period, including 78 open and 48 laparoscopic resections. Baseline demographic and clinical characteristics are presented in Table 1.

**Table 1:** Baseline demographic and clinical characteristics of patients undergoing major hepatectomy for benign liver disease, according to surgical approach (open vs. laparoscopic)

	Open	Laparoscopy	*p*
	(*n* = 78)	(*n* = 48)	
Age (years), median(IQR)	44.5(34.2–58.6)	47.1(32.2–54.9)	0.837
BMI (kg/m²), median(IQR)	25.3(21.7–27.2)	27.0(24.5–29.9)	**0.026**
Gender			
Female, n(%)	62(79.5)	42(87.5)	0.182
Male, n(%)	16(20.5)	6(12.5)	
Country			
France, n(%)	11(14.1)	18(37.5)	**0.003**
Brazil, n(%)	67(85.9)	30(62.5)	
Diabetes, n(%)	6(9.0)	4(12.9)	0.392
Hypertension, n(%)	21(30.4)	12(33.3)	0.464
Dyslipidemia, n(%)	3(3.8)	3(6.3)	0.302
Cardiopathy, n(%)	1(1.5)	1(3.3)	0.525
Pneumopathy, n(%)	2(3.0)	2(6.7)	0.364
Nephropathy, n(%)	1(1.5)	0(0.0)	0.691
Hypothyroidism, n(%)	4(6.0)	2(6.7)	0.606
ASA			
I, n(%)	33(42.3)	27(56.3)	0.260
II, n(%)	39(50.0)	17(35.4)	
III, n(%)	6(7.7)	4(8.3)	
Number of lesions			
Solitary lesion, n(%)	13(72.2)	10(83.3)	0.403
Multiple lesion, n(%)	5(27.8)	2(16.7)	
Largest lesions (mm), median (IQR)	107.5(90.0-140.0)	78.0(63.0-120.0)	0.051

ASA, American Society of Anesthesiologists; BMI, body mass index; IQR = interquartile range (P25–P75)

### Patient characteristics

The two groups were comparable in age (median 44.5 vs. 47.1 years; *p* = 0.837) and gender distribution, with a predominance of women in both cohorts. BMI was significantly higher in the laparoscopic group (27.0 vs. 25.3 kg/m²; *p* = 0.026). Country of origin differed between groups, with more Brazilian patients undergoing open surgery and more French patients undergoing laparoscopic resections (*p* = 0.003). Comorbidities and ASA classification were similarly distributed and showed no statistically significant differences. Lesions were predominantly solitary in both groups, and although median tumor size was larger in the open cohort, it did not reach statistical significance (107.5 vs. 78.0 mm; *p* = 0.051).

### Pathological findings

Final histopathological diagnoses are summarized in Table 2.

**Table 2:** Definitive anatomopathological diagnoses of resected liver lesions according to surgical approach (Open vs. Laparoscopic)

	Open (*n* = 78)		Laparoscopy (*n* = 48)		*p*
	*n*	% (95%CI)	*n*	% (95%CI)	
Mucinous cystic neoplasm	21	26.9 (17.5–38.1)	9	18.8 (9.1–32.0)	0.406
Hepatocellular adenoma	13	16.7 (9.1–26.8)	15	31.3 (19.5–45.6)	0.091
Intrahepatic lithiasis	19	24.4 (15.6–35.1)	7	14.6 (6.0–27.8)	0.276
Simple hepatic cyst	5	6.4 (2.1–14.2)	7	14.6 (6.0–27.8)	0.228
Benign biliary stricture	9	11.5 (5.4–20.8)	1	2.1 (0.1–10.9)	0.088
Hemangioma	7	9.0 (3.7–17.5)	1	2.1 (0.1–10.9)	0.154
Focal nodular hyperplasia	1	1.3 (0.0–6.9)	5	10.4 (3.4–22.7)	**0.030**
Angiomyolipoma	2	2.6 (0.3–9.1)	1	2.1 (0.1–10.9)	1.000
Hemangioendothelioma	1	1.3 (0.0–6.9)	1	2.1 (0.1–10.9)	1.000
Hamartoma	0	0.0 (0.0–4.6)	1	2.1 (0.1–10.9)	0.381

95% CI, confidence interval

Mucinous cystic neoplasm (MCN), hepatocellular adenoma (HCA), and intrahepatic lithiasis were the most frequent etiologies across the cohort. The distribution of diagnoses varied between groups: focal nodular hyperplasia (FNH) was significantly more prevalent in the laparoscopic cohort (10.4% vs. 1.3%; *p* = 0.030), whereas benign biliary strictures and intrahepatic lithiasis were more common in open resections, consistent with their technically demanding nature. There were no significant differences in the frequency of MCN or HCA between approaches.

### Intra-operative results

Intraoperative parameters are shown in Table 3.

**Table 3:** Intraoperative characteristics according to surgical approach (Open vs. Laparoscopic)

	Open	Laparoscopy	*p*
	(*n* = 78)	(*n* = 48)	
Right hepatectomy, n (%)	42 (53.8)	24 (50.0)	0.572
Left hepatectomy, n (%)	36 (46.2)	24 (50.0)	0.440
Pringle maneuver			
No, n(%)	43 (55.1)	18 (37.5)	0.165
Hemi-Pringle, n(%)	3 (3.8)	2 (4.2)	
Pringle, n(%)	32 (41.0)	28 (58.3)	
Pringle maneuver time (min), median(IQR)	29.0 (15.0–45.0)	48.0 (35.0–80.0)	**0.002**
Operative time (min), median(IQR)	330.0 (260.0–430.0)	312.5 (200.0–400.0)	0.068
Blood loss (ml), median(IQR)	400.0 (200.0–800.0)	150.0 (20.0–280.0)	**< 0.001**
Intraoperative transfusion, n(%)	30 (38.5)	4 (8.3)	**0.001**

IQR = interquartile range (P25–P75)

The distribution of right and left hepatectomies did not differ significantly between groups. The Pringle maneuver was used more frequently in the laparoscopic cohort (58.3% vs. 41.0%), and the duration of inflow occlusion was significantly longer (median 48.0 vs. 29.0 min; *p* = 0.002).

Laparoscopic surgery was associated with markedly reduced blood loss (median 150 vs. 400 mL; *p* < 0.001) and a significantly lower rate of intraoperative blood transfusion (8.3% vs. 38.5%; *p* = 0.001). Operative time was comparable (312.5 vs. 330.0 min; *p* = 0.068). Four laparoscopic cases (8.3%) required conversion, all due to intraoperative bleeding.

### Post-operative outcomes

Postoperative complications, ICU metrics, and length of stay are detailed in Table 4.

**Table 4:** Postoperative complications, ICU admission and hospital stay according to surgical approach (Open vs. Laparoscopic)

	Open (n=78)	Laparoscopy (n=48)	p
Clavien–Dindo classification (grouped)			
With complications, n(%)	33 (42.3)	13 (27.1)	0.091
Minor complications, n(%)	17 (21.8)	7 (14.6)	0.359
Moderate complications, n(%)	10 (12.8)	4 (8.3)	0.565
Severe complications, n(%)	6 (7.7)	2 (4.2)	0.709
Specific complications			
Aponeurotic suture dehiscence, n (%)	1 (1.3)	0 (0)	0.993
Intra-abdominal bleeding, n (%)	1 (1.3)	0 (0)	0.993
Bile leakage, n (%)	16 (20.5)	2 (4.2)	**0.016**
Ascites, n (%)	1 (1.3)	1 (2.1)	0.989
Intra-abdominal abscess (or collection), n (%)	3 (3.8)	4 (8.3)	0.426
Pulmonary embolism, n (%)	1 (1.3)	2 (4.2)	0.557
Myocardial infarction, n (%)	0 (0)	1 (2.1)	0.381
Prolonged ileus (NGT > 3 days), n (%)	4 (5.1)	0(0)	0.297
Pneumonia, n (%)	1 (1.3)	3 (6.2)	0.154
Hepatic failure, n (%)	1 (1.3)	0 (0)	0.993
Acute kidney injury (AKI), n (%)	4 (5.1)	0 (0)	0.297
ICU admission, n(%)	64 (82.1)	36 (75)	0.371
ICU time (days), median (IQR)	2.0 (1.0-3.0)	1.0 (1.0-2.0)	0.107
Length of hospital stay (days), median (IQR)	7.0 (6.0-11.0)	5.0 (4.0-6.0)	**<0.001**

ICU, intensive care unit; BMI, IQR = interquartile range (P25–P75)

Overall complication rates were lower in the laparoscopic group (27.1% vs. 42.3%), although the difference did not reach statistical significance (*p* = 0.091). The distribution of minor (Clavien I–II), moderate (III), and severe (IV) complications was similar between groups.

Bile leakage occurred significantly more often after open surgery (20.5% vs. 4.2%; *p* = 0.016). Among the 18 cases of bile leakage, 13 were classified as ISGLS grade A, including both cases in the laparoscopic group. Two were classified as grade B and were successfully managed with percutaneous drainage, whereas three were grade C and required surgical reoperation. Notably, all grade B and C leaks occurred after open resection. Other complications, including ascites, prolonged ileus, AKI, intra-abdominal abscess, and cardiopulmonary events, were more frequent in the open group, although without statistical significance.

ICU admission rates were similar (82.1% vs. 75.0%; *p* = 0.371). ICU stay tended to be shorter after laparoscopy (1.0 vs. 2.0 days; *p* = 0.107). Total hospital stay was significantly reduced in the laparoscopic group (median 5.0 vs. 7.0 days; *p* < 0.001). No 90-day mortality occurred in either group.

Within 30 days after discharge, 7 patients (5.6%) required hospital readmission. The causes included intra-abdominal abscess or fluid collection (*n* = 4), bile leakage (*n* = 2), and pulmonary embolism (*n* = 1). Six readmissions occurred after open surgery (7.7%), whereas one occurred after laparoscopic surgery (2.1%), although this difference did not reach statistical significance (*p* = 0.25).

Multivariate logistic regression **(**Table 5**)** demonstrated that the laparoscopic approach was an independent protective factor against intraoperative blood transfusion (OR 0.096; 95% CI 0.011–0.862; *p* = 0.036), after adjusting for BMI, operative time, Pringle time, and type of hepatectomy.

**Table 5:** Logistic regression models predicting intraoperative transfusion and hospital stay by surgical approach (open vs. laparoscopic)

	B	p	OR	95% CI
Intraoperative blood transfusion (Yes/No)				
Surgical approach (laparoscopic vs. open)	–2.342	**0.036**	0.096	0.011 – 0.862
BMI (kg/m²)	0.112	0.376	1.119	0.873 – 1.435
Total Pringle time (min)	–0.019	0.490	0.981	0.930 – 1.035
Type of hepatectomy (left vs. right)	–1.465	0.124	0.231	0.036 – 1.492
Operative time (min)	0.001	0.806	1.001	0.994 – 1.008
Prolonged hospital stay (≥7 days vs. ≤6 days)				
Surgical approach (laparoscopic vs. open)	–2.489	**0.036**	0.083	0.008 – 0.847
BMI (kg/m²)	0.003	0.980	1.003	0.795 – 1.266
Total Pringle time (min)	0.015	0.523	1.015	0.970 – 1.063
Operative time (min)	0.002	0.708	1.002	0.993 – 1.010
Gender (male vs. female)	0.271	0.831	1.311	0.109 – 15.733
Type of hepatectomy (left vs. right)	–1.537	0.096	0.215	0.035 – 1.317

The results of the models were expressed as regression coefficient (B), p-value, odds ratio (OR), and corresponding 95% confidence intervals (95% CI). BMI, body mass index

Similarly, laparoscopic surgery was independently associated with a lower likelihood of prolonged hospital stay, which translated into a greater probability of early discharge (OR 0.083; 95% CI 0.008–0.847; *p* = 0.036). No other covariates were significantly associated with transfusion or length of stay.

## Discussion

This international bicentric study provides comparative data on laparoscopic and open major hepatectomy for benign liver disease in two high-volume hepatobiliary centers. While laparoscopic liver resection (LLR) is well established for minor peripheral lesions, evidence addressing major resections in benign conditions remains relatively limited, with most published series focusing on malignant indications [9, 22]. The present analysis adds international comparative data specifically addressing benign disease.

Benign liver disease predominantly affects young and otherwise healthy patients, underscoring the importance of careful risk–benefit assessment before proposing major hepatectomy [23–25]. In line with previous reports, the present cohort consisted largely of low-risk patients (ASA I–II) with few comorbidities, a profile that likely contributed to the overall favorable perioperative outcomes observed.

Etiological distribution differed between groups in expected ways: focal nodular hyperplasia, usually small, superficial lesions in young individuals, was more frequently treated laparoscopically (*p* = 0.030), while inflammatory biliary conditions such as intrahepatic lithiasis and benign biliary strictures were more common in the open cohort, reflecting their higher technical complexity [25]. Tumor size tended to be smaller in the laparoscopic cohort, consistent with known patterns of case selection, although this difference did not reach statistical significance. Recent data show that LLR can safely manage even large benign lesions when performed by experienced teams, supporting the progressive expansion of indications [8, 9].

Multiple lesions were slightly more frequent in the open surgery group, likely reflecting surgeon preference and the perceived technical complexity of managing multifocal disease laparoscopically, particularly during earlier phases of institutional experience. However, this difference did not reach statistical significance.

In the present study, laparoscopic major hepatectomy demonstrated consistent perioperative advantages, including reduced blood loss, lower transfusion rates, and shorter hospital stay. These findings are concordant with those reported in large multicenter series and meta-analyses [26]. Improved hemostasis during LLR has been attributed to enhanced visualization, modern energy devices, pneumoperitoneum-related venous compression, and controlled vascular division [27, 28]. The more frequent use and longer duration of the Pringle maneuver in the laparoscopic group may also have contributed to reduced blood loss; importantly, intermittent inflow occlusion has been shown to be safe for the liver remnant [21].

Overall morbidity did not differ significantly between approaches. However, specific complications, including bile leakage, prolonged ileus, and acute kidney injury, were less frequent after laparoscopy. Prolonged ileus and acute kidney injury occurred exclusively in the open group, findings consistent with previous reports associating open surgery with greater visceral manipulation, inflammatory stress, and delayed gastrointestinal recovery [17, 18]. Post-hepatectomy liver failure was rare in this cohort, likely reflecting the favorable baseline clinical profile of the patients. Laboratory markers such as transient elevations in serum transaminases may indicate ischemia–reperfusion injury but do not necessarily correlate with clinically significant hepatic dysfunction [19]. Therefore, validated clinical criteria, such as the definition proposed by the ISGLS, were used to assess postoperative liver failure

The incidence of bile leakage was significantly lower after laparoscopic hepatectomy. All leaks observed in the laparoscopic group were mild (ISGLS grade A), whereas clinically significant events (grade B or C) occurred exclusively after open resections and required interventional management. These findings are consistent with those reported in international multicenter analyses and propensity score–matched studies [20, 26]. The observed differences may reflect both technical advantages of the laparoscopic approach and the greater complexity of biliary conditions more frequently managed through open surgery.

The conversion rate of 8.3% observed in this series was within the lower range of rates reported in major international studies [29]. Conversions were primarily related to intraoperative bleeding and occurred predominantly during the early phase of the experience, consistent with the recognized learning curve for major laparoscopic hepatectomy, estimated at 45–90 procedures [11, 30, 31]. Although the long study period introduces variability related to evolving expertise and technology, it also reflects the real-world maturation of laparoscopic liver surgery programs.

Although lesions tended to be larger in the open surgery group, this difference did not reach statistical significance. Importantly, multivariate analysis adjusting for relevant intraoperative variables confirmed the laparoscopic approach as an independent protective factor against intraoperative transfusion and prolonged hospitalization, supporting the robustness of the observed benefits. Therefore, the reduced transfusion requirement observed in the laparoscopic group is unlikely to be explained solely by lesion size or inflow occlusion time and may also reflect technical advantages of the minimally invasive approach.

This study has several limitations inherent to its retrospective design. First, the absence of randomization may have introduced selection bias in the choice of surgical approach. In addition, although both participating institutions are high-volume hepatobiliary centers, differences in case selection and local expertise may have influenced the distribution of surgical approaches, as reflected by the unequal representation of Brazilian and French cases. The extended study period also encompasses the progressive evolution of laparoscopic liver surgery, potentially introducing temporal variability related to increasing surgical experience and advances in technology. Finally, the analysis focused primarily on short-term perioperative outcomes and did not address long-term clinical results, which were beyond the scope of the present study. Nevertheless, strict inclusion and exclusion criteria were applied, and baseline demographic and clinical characteristics were broadly comparable between groups, allowing a meaningful comparison of perioperative outcomes, although residual confounding related to case selection cannot be completely excluded. The absence of postoperative mortality, however, underscores the high level of expertise at the participating centers.

Overall, these findings support laparoscopic major hepatectomy as a feasible, safe, and reproducible approach for benign liver disease when performed in experienced centers, with consistent benefits across institutions. LLR offers meaningful clinical advantages, including lower blood loss, reduced transfusion requirements, shorter hospitalization, and fewer biliary and systemic complications, without increasing operative time or mortality. Prospective multicenter studies with larger cohorts are warranted to refine patient selection and validate these results.

## Conclusion

In this international bicentric cohort, laparoscopic major hepatectomy was associated with favorable short-term outcomes compared with open surgery, including reduced intraoperative blood loss, lower transfusion rates, shorter hospitalization, and fewer biliary complications, without increases in operative time or mortality. When performed by experienced hepatobiliary surgeons in high-volume centers, LLR represents a safe and effective surgical approach for selected patients with benign liver disease.

## Data Availability

Data are available upon reasonable request from the corresponding author.

## Declarations

### Clinical trial registration

N/A
